# A phase II clinical trial of adoptive transfer of haploidentical natural killer cells for consolidation therapy of pediatric acute myeloid leukemia

**DOI:** 10.1186/s40425-019-0564-6

**Published:** 2019-03-20

**Authors:** Rosa Nguyen, Huiyun Wu, Stanley Pounds, Hiroto Inaba, Raul C. Ribeiro, David Cullins, Barbara Rooney, Teresa Bell, Norman J. Lacayo, Kenneth Heym, Barbara Degar, Deborah Schiff, William E. Janssen, Brandon Triplett, Ching-Hon Pui, Wing Leung, Jeffrey E. Rubnitz

**Affiliations:** 1 Department of Oncology, St. Jude Children’s Hospital, 262 Danny Thomas Place, Memphis, TN 38105 USA; 2 Department of Biostatistics, St. Jude Children’s Hospital, Memphis, TN USA; 3 Department of Bone Marrow Transplantation and Cellular Therapy, St. Jude Children’s Hospital, Memphis, TN USA; 4 Lucile Packard Children’s Hospital and Stanford Cancer Center, Palo Alto, CA USA; 5 Cook Children’s Medical Center, Fort Worth, TX USA; 6 Dana-Farber Cancer Institute, Boston, MA USA; 7 Rady Children’s Hospital, San Diego, CA USA; 8 Human Applications Laboratory, St. Jude Children’s Research Hospital, Memphis, TN USA

**Keywords:** Acute myeloid leukemia, Child, Clinical trial, Natural killer cells

## Abstract

**Abstract:**

Consolidation therapies for children with intermediate- or high-risk acute myeloid leukemia (AML) are urgently needed to achieve higher cure rates while limiting therapy-related toxicities. We determined if adoptive transfer of natural killer (NK) cells from haploidentical killer immunoglobulin–like receptor (KIR)–human leukocyte antigen (HLA)-mismatched donors may prolong event-free survival in children with intermediate-risk AML who were in first complete remission after chemotherapy. Patients received cyclophosphamide (Day − 7), fludarabine (Days − 6 through − 2), and subcutaneous interleukin-2 (Days − 1, 1, 3, 5, 7, and 9). Purified, unmanipulated NK cells were infused on Day 0, and NK cell chimerism and phenotyping from peripheral blood were performed on Days 7, 14, 21, and 28. As primary endpoint, the event-free survival was compared to a cohort of 55 patients who completed chemotherapy and were in first complete remission but did not receive NK cells. Donor NK cell kinetics were determined as secondary endpoints. Twenty-one patients (median age at diagnosis, 6.0 years [range, 0.1–15.3 years]) received a median of 12.5 × 10^6^ NK cells/kg (range, 3.6–62.2 × 10^6^ cells/kg) without major side effects. All but 3 demonstrated transient engraftment with donor NK cells (median peak donor chimerism, 4% [range, 0–43%]). KIR–HLA-mismatched NK cells expanded in 17 patients (81%) and contracted in 4 (19%). However, adoptive transfer of NK cells did not decrease the cumulative incidence of relapse (0.393 [95% confidence interval: 0.182–0.599] vs. 0.35 [0.209–0.495]; *P =* .556) and did not improve event-free (60.7 ± 10.9% vs. 69.1 ± 6.8%; *P* = .553) or overall survival (84.2 ± 8.5% vs. 79.1 ± 6.6%; *P* = .663) over chemotherapy alone. The lack of benefit may result from insufficient numbers and limited persistence of alloreactive donor NK cells but does not preclude its potential usefulness during other phases of therapy, or in combination with other immunotherapeutic agents.

**Trial registration:**

www.clinicaltrials.gov, NCT00703820. Registered 24 June 2008.

## Introduction

Approximately one-third of children with acute myeloid leukemia (AML) experience disease relapse despite risk-adapted intensive multiagent chemotherapy with or without hematopoietic cell transplantation (HCT) [1]. Particularly improved consolidation therapies for children with intermediate- or high-risk cytogenetic features are urgently needed to achieve higher cure rates while limiting therapy-related toxicities.

The first evidence for the therapeutic effectiveness of natural killer (NK) cells in AML was established in a study of 57 adult patients, in which none of the 20 individuals who received haploidentical cell transplantation from a killer immunoglobulin–like receptor (KIR)–human leukocyte antigen (HLA)-mismatched donor experienced disease relapse [2]. This finding has been confirmed in both adults and pediatrics [3, 4]. NK cells play an important role in infection and cancer prevention [5]. Unlike cytotoxic T cells, they do not require prior sensitization to eliminate tumor cells but mediate cytotoxicity through complex interactions between NK cell surface receptors and their cognate ligands expressed on their respective target cells [6]. The inhibitory KIRs 2DL1 (CD158a), 2DL2/3 (CD158b), and 3DL1 (CD158e1) are such surface receptors and allow adoptively transferred donor NK cells to exert potent antileukemic effects if the cognate HLA class I ligands are absent in the recipients [3]. This potency has been demonstrated in preclinical experiments [2], as well as in clinical studies, without causing graft-versus-host disease [3, 4]. This has led to the development of two therapeutic approaches to harness the antileukemic effect of alloreactive NK cells for patients with AML. Patients may receive myeloablative chemotherapy followed by allogeneic HCT to induce permanent engraftment of donor KIR–HLA-mismatched NK cells [3, 4]. Patients may alternatively receive lympho-depleting chemotherapy followed by adoptive transfer of purified NK cells from an alloreactive donor, resulting in homeostatic lymphocyte proliferation with transient donor NK cell expansion in recipients [7].

Previously, we performed the first study of adoptive transfer of haploidentical NK cells in children with AML, all of whom were in complete remission [8]. The NK cell purification process was deemed feasible, and NK cell infusion was tolerated by all participants, leading to in vivo expansion of KIR–HLA-mismatched NK cells in 9 of 10 patients. Additionally, all patients remained in complete remission.

To determine whether haploidentical NK cell therapy prolongs event-free survival in children with intermediate-risk AML, we conducted a phase 2 study in which we administered a low-intensity lympho-depleting regimen followed by infusion of highly purified KIR–HLA-mismatched NK cells from haploidentical donors. Donor NK cell kinetics were determined as secondary endpoints.

## Patients and methods

### Patient and donor eligibility

Children with intermediate-risk AML who were in first complete remission after completion of 4 or 5 courses of chemotherapy as part of the randomized controlled phase 3 clinical trial (NCT00703820) and who had a KIR–-HLA-mismatched parent were eligible for adoptive transfer of haploidentical NK cells. No other criteria were considered for eligibility. Intermediate risk was defined by (1) presence of core binding factor leukemia with minimal residual disease of ≥ 0.1% but < 5% on Day 22 of therapy or rising levels of fusion transcript, (2) presence of *FLT3-ITD* fusion and negative minimal residual disease on Day 22 of therapy, or (3) absence of low-risk or high-risk features as previously defined [9]. Histocompatibility testing, including HLA and KIR genotyping, was performed for every patient and donor in a laboratory at St. Jude Children’s Research Hospital. A KIR–HLA mismatch was defined by a lack of the cognate HLA class I molecule in patients that corresponded with the respective KIR identified in NK cell donors. CD158a (KIR2DL1) was determined to be specific for HLA-C2 allotypes with lysine at position 80 (HLA-CLys80), CD158b1/b2 (KIR2DL2/2DL3) for B*4601 and HLA-C1 allotypes with asparagine at position 80 (HLA-CAsn80), and CD158e1 (KIR3DL1) for HLA-B allotypes expressing the Bw4 epitope (HLA-Bw4) [10]. Potential donors underwent clearance procedures to determine eligibility [11]. The study was approved by our institutional review board, and informed consent was obtained from parents or guardians, and assent from the patients, as appropriate. Patients were monitored for 45 days after NK cell infusion for absolute neutrophil (≥ 500 cells/μL and rising) and platelet count recovery (≥ 20,000 cells/μL and rising), graft-versus-host disease, and adverse events ≥ grade 3 [12, 13].

Patients with intermediate-risk AML who did not receive NK therapy but had completed at least 4 courses of chemotherapy as part of the randomized controlled phase 3 clinical trial (NCT00703820) were used as control cohort for survival analysis.

### NK cell collection and treatment regimen

Patients with rising absolute neutrophil counts ≥ 300/μL and platelet counts ≥ 30,000/μL received the following conditioning regimen: Cyclophosphamide (60 mg/kg) was intravenously (IV) administered on Day − 7, and fludarabine (25 mg/m^2^ per day) was IV administered on Days − 6 through − 2. Patients received interleukin-2 (1 × 10^6^ units/m^2^) subcutaneously on Days − 1, 1, 3, 5, 7, and 9. Donors underwent apheresis on Day − 1, and mononuclear cells were purified in a two-step procedure for CD56^+^/CD3^−^ NK cells with the CliniMACS system (Miltenyi Biotec, Woburn, MA) as preciously described [8], allowing for a CD56^−^/CD3^+^ cell dose of < 0.05 × 10^6^ cells/kg. Purified, unmanipulated NK cells were infused on Day 0 at a desired dose of > 2 × 10^6^ CD56^+^/CD3^−^ cells/kg recipient body weight.

### Correlative biology studies

NK cell chimerism and phenotyping from peripheral blood were performed on Days 7, 14, 21, and 28 after NK cell infusion. While blood for phenotype analysis was collected on time, there was up to a 48-h deviation from these time points when blood was sampled for NK cell chimerism studies due to clinical care considerations (e.g. maximum blood draw limit). Chimerism studies of NK cells purified by fluorescence-activated cell sorting were performed by standard variable number tandem repeats techniques [14]. NK cell phenotyping was determined by flow cytometric measurement of cell surface receptor expression with the following antibodies: CD158a detection by clone 143,211, CD158b by CH-L (R&D systems, Minneapolis, MN), CD158e1 by DX9 (BD Biosciences, San Jose, CA), NKG2A by Z199, NKp30 by Z25, NKp44 by Z231, NKp46 by BAB281 (Beckman Coulter, Indianapolis, IN), and NKG2D by 1D11 (BD Biosciences). We defined alloreactive NK cells as the fraction of CD56^+^/CD3^−^ cells that expressed the HLA-mismatched KIR by flow cytometry analysis but was negative for the NKG2A and KIRs corresponding with patient expressed HLAs. A rise in donor NK cell chimerism or KIR–HLA mismatched NK cell number after Day 7 was defined as NK cell expansion.

### Statistics

Summary statistics were used to report clinical information. Both trend effects and time points difference in mean white blood cell and NK cell counts over time were examined by using linear mixed-effects models. Spearman correlation was performed to test the relation between NK cell chimerisms and NK cell dose. In analysis of event-free survival, an event was defined as relapse or death. Survival analysis was performed by comparing patients who received NK cell therapy with those who also had intermediate-risk AML and did not receive NK therapy but had completed at least 4 courses of chemotherapy in the AML08 clinical trial (NCT00703820). A linear Cox hazard proportional regression model was applied to logarithmically transformed NK cell receptor expression and NK cell counts to assess their association with survival. Data were analyzed using R.

## Results

### Clinical characteristics of study cohort

Twenty-one patients (11 males [52%]) with a median age of 6.0 years (range, 0.1–15.3 years) at diagnosis received NK cell therapy. Sixteen (76%) patients completed 4 courses and 5 (24%) completed 5 courses of chemotherapy. All patients were negative for minimal residual disease (defined by < 0.1% leukemia cells) at the time of NK cell infusion. Patient demographic, hematologic, and engraftment characteristics are summarized in Table 1. The median duration of time from day 1 of the last cycle of chemotherapy to infusion of NK cells was 53 days (range, 31–111 days) and 10 days (range, 3–57 days) from the previous bone marrow aspirate obtained after completion of chemotherapy. The patients received a median of 12.5 × 10^6^ NK cells/kg (range, 3.6–62.2 × 10^6^ cells/kg). Every patient tolerated and completed the preparative regimen and NK cell infusion with only 1 nonhematologic grade 3 adverse event (extravasation skin injury from cyclophosphamide). Of the 21 patients treated, 20 (95%) developed ≥ grade 3 neutropenia, and 5 (24%) developed grade ≥ 3 thrombocytopenia. All patients but 1 had absolute neutrophil (median time to recovery, 13 days [7–45]) and platelet count recovery (median time to recovery, 33 days [9–52]) within 45 days after NK cell infusion and none had opportunistic infections or notable bleeding. Graft-versus-host disease did not occur in any patients.

**Table 1:** Clinical Characteristics of Patients Who Received NK Cell Therapy

UPN	Characteristics at Diagnosis			HLA Status		Donor KIR– Recipient HLA Mismatch	Peak NK Cell Chimerism		Adoptive NK Cell Transfer	Days from NK cell transplant to relapse
	Age at diagnosis (Years)	Sex	Karyotype	Bw	C		%	Day	NK Cells^−^ (10^6^/kg)	
1	1.1	M	t(11;19)	6/6	C1/C1	CD158a, CD158e1	0	7	16.5	–
2	1.6	F	+ 4,t(6;11),+der(6)t(6;11),+ 8,+ 13,+ 19	4/6	C2/C2	CD158b	1	14	11.5	629
3	2.1	M	t(9;11),+X,+ 7,+ 8	4/6	C2/C2	CD158b	2	6	12.4	–
4	10.2	F	46,XX	4/6	C2/C2	CD158b	4	12	8.2	527
5	1.2	F	t(1;22),+ 2,+ 8,+ 21,+der(22)t(1;22)	4/6	C1/C1	CD158a	23	7	46.9	617
6	15.4	F	46,XX	6/6	C1/C2	CD158e1	8	13	3.6	–
7	1.2	F	t(11;19),t(12;19)	4/6	C2/C2	CD158b	2	8	36.9	–
8	0.1	F	N/A	6/6	C1/C2	CD158e1	43	14	44.3	–
9	0.5	F	der(10)	4/6	C1/C1	CD158a	15	7	26.8	–
10	4.5	M	46,XY	6/6	C1/C1	CD158a, CD158e1	6	7	14.8	300^†^
11	2.1	M	t(1;11),+ 9	6/6	C1/C1	CD158a, CD158e1	2	7	25.7	–
12	13.2	F	+ 8,t(11;19),+ 17,+ 19	6/6	C1/C1	CD158a, CD158e1	15	7	6.7	228
13	15.3	F	+ 21	4/6	C1/C1	CD158a	14	12	5.2	–
14	12.4	M	t(1;2),del(9),-12,t(17;20),der(18)	4/6	C1/C1	CD158a	3	7	8.8	186^†^
15	1.6	M	+ 8,t(9;11;14)	4/6	C2/C2	CD158b	0	7	12.5	–
16	14.3	F	ins(10;11),ins(17;11)	4/6	C1/C1	CD158a	22	7	5.7	231^†^
17	0.7	M	t(5;6)	4/6	C2/C2	CD158b	32	21	42.4	–
18	3.0	M	46,XY	4/6	C1/C1	CD158a	0	7	62.6	–
19	4.0	M	46,XY	4/6	C1/C1	CD158a	4	7	12.4	–
20	12.2	M	+X,+ 9,+ 11,+ 14,+ 20	6/6	C1/C2	CD158e1	1	7	9.0	367
21	9.2	M	46,XY	6/6	C1/C1	CD158a, CD158e1	10	7	19.3	–

*Abbreviations*: *CNS* central nervous system, *HLA* human leukocyte antigen, *KIR* killer immunoglobulin-like receptor, *N/A* not available, *NK* natural killer, *UPN* unique patient number. ^†^ Deceased

### NK cell kinetics after adoptive transfer

**Fig. 1:**
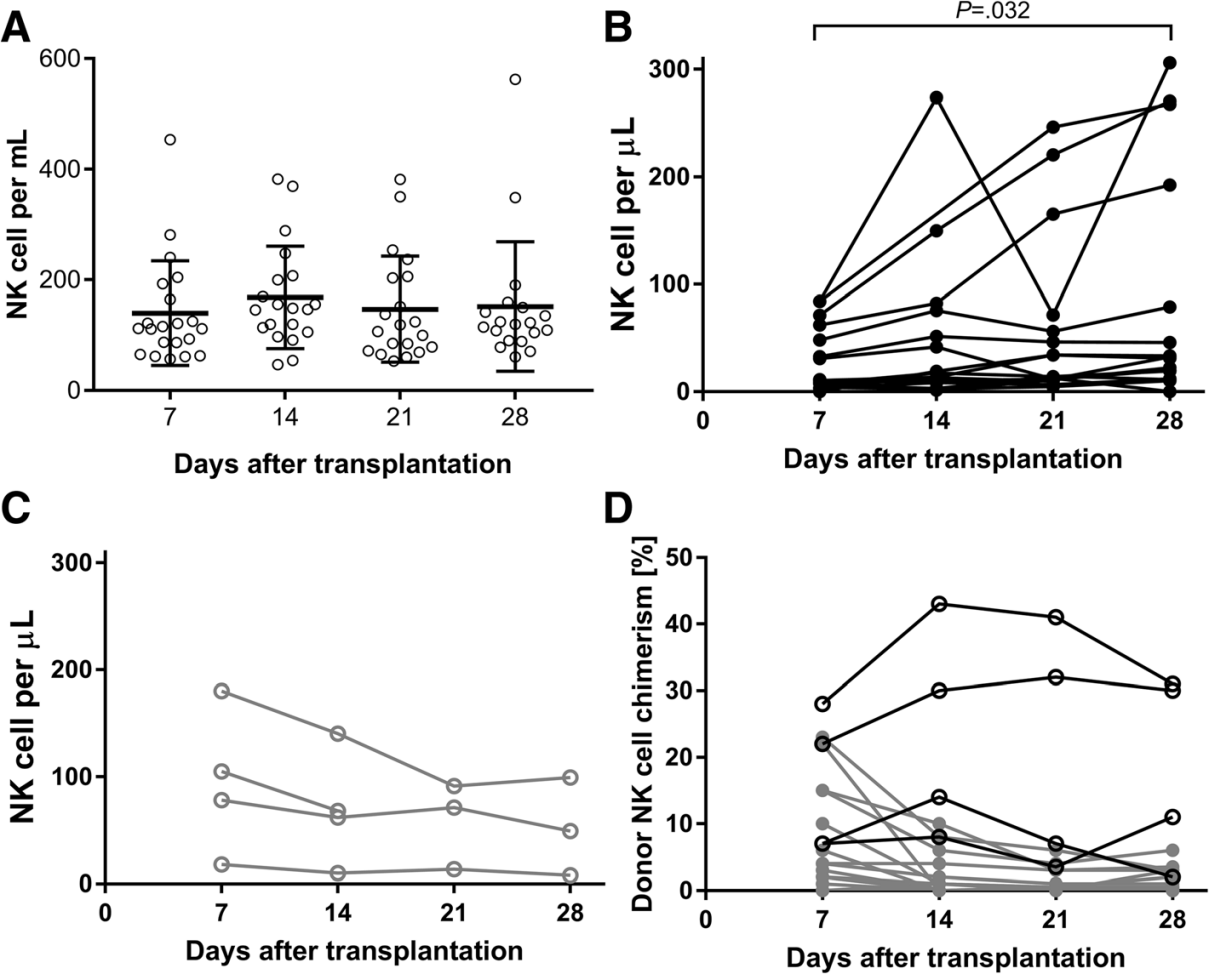
Average natural killer (NK) cell engraftment (**a**) in peripheral blood plotted over time. KIR–HLA-mismatched NK cells expanded over time in 17 patients (**b**) and contracted in 4 patients (**c**). Donor NK cell chimerism was detected in 18 patients (**d**, gray line), of which 4 were noted to have rising levels over time (black line)

On Day 7 after NK cell infusion, the median absolute blood NK cell count was 112 cells/μL (range, 57–454 cells/μL), which remained stable with a median of 148 cells/μL (range, 47–382 cells/μL) on Day 14, 113 cells/μL on Day 21 (range, 53–382 cells/μL), and 120 cells/μL on Day 28 (range, 61–562 cells/μL; Fig. 1a). Although white blood cell counts continuously increased after NK cell infusion (*P =* .038), the NK cell numbers remained stable during that period (*P >* .05). The average number of alloreactive NK cells increased from 41 cells/μL (range, 0–180 cells/μL) on Day 7 to 53 cells/μL (range, 2–274 cells/μL) on Day 14, 57 cells/μL (range, 5–246 cells/μL) on Day 21, and 79 cells/μL (range, 0–306 cells/μL; *P =* .032) on Day 28. The fraction of KIR–HLA-mismatched, alloreactive NK cells expanded in 17 patients (Fig. 1b) and contracted in 4 patients (Fig. 1c). We detected donor NK cell chimerism in a total of 18 patients, 4 of whom were noted to have rising NK cell levels over time (Fig. 1d). The median peak donor NK cell chimerism was 4% (range, 0–43%; Table 1). Eleven of 18 patients (61%) had persistent levels above the threshold of 1% at 4 weeks after NK cell infusion (median, 2%; range, 1–30%). The level of alloreactive NK cells in the peripheral blood or donor NK cell chimerism was not correlated with the infused NK cell dose (*P* > .05).

### Survival analysis

**Fig. 2:**
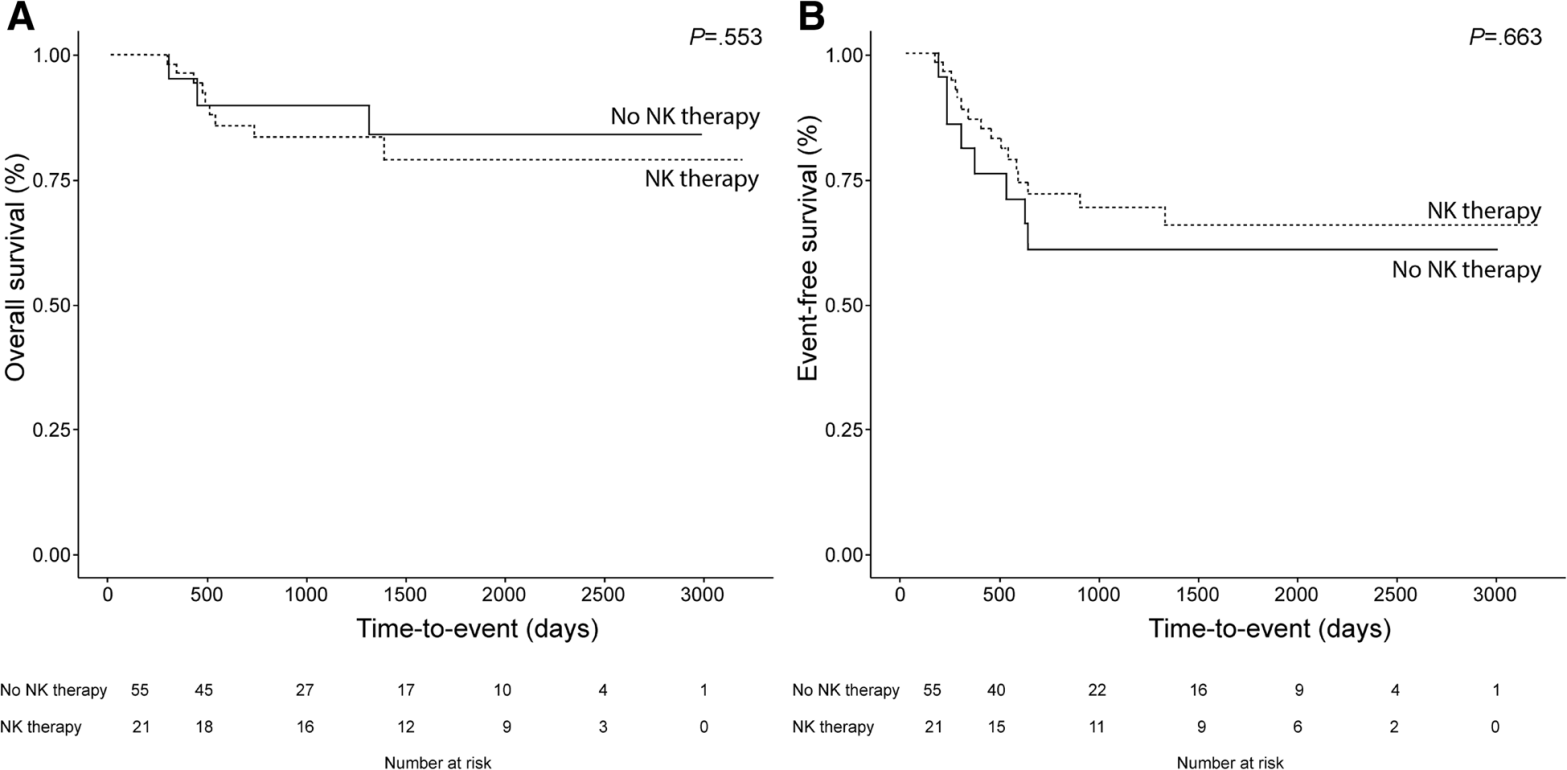
Kaplan-Meier survival curves comparing the overall survival (**a**) and event-free survival (**b**) of 21 patients with NK cell infusion (dashed line) with that of 53 patients who completed 4 courses of chemotherapy but did not receive NK cell infusion (solid line)

Basic demographic and hematologic characteristics of the study cohort and controls are summarized in Table 2. Of the 8 patients (38%) who experienced relapse after adoptive transfer of NK cells, 3 (14%) died of the disease. The median follow-up time for the entire cohort was 1698 days, and relapse occurred between 186 and 629 days after NK cell infusion. NK cell infusion did not improve the cumulative incidence of relapse (0.393 [95% confidence interval: 0.182–0.599] vs. 0.35 [0.209–0.495]; *P =* .556), event-free survival (60.7 ± 10.9% vs. 69.1 ± 6.8%; *P* = .553) or overall survival (84.2 ± 8.5% vs. 79.1 ± 6.6%; *P* = .663) compared to chemotherapy alone (Fig. 2). Our calculations indicated that survival distributions would have been statistically significantly different between the treated group and the control group if all patients in the treated group had been free of an event (100% vs 69.1%). Event-free survival but not overall survival was associated with the expression of CD158a (*P =* .028), CD158b (*P =* .021), and NKG2D (*P =* .017), as well as with the NK cell count (*P =* .007) on Day 7. The number of alloreactive NK cells was not associated with event-free or overall survival at any time point.

**Table 2:** Demographic and hematologic characteristics of study and control cohort

	*N* = 21	*N* = 55	*P* value
Age at diagnosis (mean [range])	1.2 (3.0–12.2)	8.5 (0.1–19.4)	0.181
Gender, male (N [%])	11 (52)	31 (56)	0.755
CNS status	N (%)	N (%)	
CNS 1	11 (52)	31 (56)	0.355
CNS 2	6 (29)	17 (31)	
CNS 3	1 (5)	6 (11)	
Missing	2 (10)	6 (11)	
FAB classification	N (%)	N (%)	
M1	1 (5)	7 (13)	0.795
M2	4 (19)	5 (9)	
M4	2 (10)	7 (13)	
M5	9 (43)	21 (38)	
M7	1 (5)	3 (5)	
Missing	2 (10)	12 (22)

## Discussion

Hematopoietic cell transplantation from KIR–HLA-mismatched donors is associated with a marked event-free survival benefit in patients with AML [2–4], but the benefit of NK cell infusion as a consolidation therapy remains unclear for children with AML. Therefore, we conducted the first phase 2 study to determine whether adoptively transferred NK cells from haploidentical and KIR–HLA mismatched donors improve event-free-survival in children with intermediate-risk AML who completed chemotherapy and were in first remission. Despite tolerability and transient engraftment of donor NK cells in most individuals, adoptive transfer of NK cells did not decrease relapse or increase survival compared to chemotherapy alone. We explain the lack of survival benefit in our cohort due to limited persistence of donor NK cells and low donor NK cell infusion dose.

The bone marrow is the main site for NK cell production, and the average life span of circulating NK cells is approximately 2 weeks [15]. Unlike other cells with adaptive immunity, mature NK cells have a very limited ability to expand and contract. Even in infectious states, such as acute mononucleosis, when overall NK cell numbers are markedly elevated, NK cell kinetics remain largely unchanged [15]. Consistent with that, most patients in our study had decreasing donor chimerism levels after 2 weeks from the time of NK cell infusion, suggesting that the kinetics of adoptively transferred NK cells follow the same pattern of decay as that of primary NK cells [15]. During HCTs, donor progenitor cells are a permanent source for alloreactive NK cells allowing for continued tumor surveillance, whereas adoptively transferred NK cells represent a limited cell pool. It is conceivable that the lack of persistence and continued immune surveillance mediated by the alloreactive NK cells resulted in failure to reduce relapse risk.

The average peak and week 4 donor NK cell chimerisms in this study were significantly lower compared to reports from our previous phase 1 trial that used an identical lymphodepleting chemotherapy regimen [8]. We believe that this discrepancy could be due to the low median number of infused NK cells (12.5 vs. 29 × 10^6^ NK cells/kg). Although we did not find a linear correlation between peak donor NK cell chimerism and infused NK cell dose, a causal relationship may still exist. Peripheral blood NK cell chimerism measurements might not accurately reflect the degree of expansion and/or persistence of allogeneic NK cells due to sequestration of these cells in the bone marrow (potentially targeting residual leukemia), or other tissues. Because the two-step process to isolate NK cells was identical in both studies, and because the proportion of young patients with low body weight was similar as well, we think that the low NK cell dose could be due to donor-related factors. Whether extending the time of IL-2 administration or alternatively administering IL-15 may lead to further expansion and donor NK cell persistence in vivo remains to be determined in future trials. Regardless, decreased numbers of adoptively transferred NK cells may have affected tumor clearance, thereby enabling the re-emergence of leukemic cell clones in patients with relapse. To support this, we found that 6 of 8 patients with disease relapse received less than the median NK cell dose. Of the 10 patients from our phase 1 trial, 6 had intermediate-risk AML and have remained relapse-free after a follow-up of approximately 32 months. Conversely, this cohort was infused with a much higher median NK cell number (29 × 10^6^ NK cells/kg) and contained only one patient (UPN 7) who received less than the median phase 2 NK cell dose.

Children in our study received purified but otherwise unmanipulated NK cells. It is conceivable that activated and expanded or chimeric antigen receptor (CAR)-expressing NK cells may have more applicability in the future. Although NK cells are more difficult to genetically modify for this purpose than T cells, they have a limited life span in vivo and potentially a more favorable toxicity profile than the latter. Artificial antigen presenting cells such as K562 with membrane-bound 4-1BBL/IL15Rα [16], IL-21 [17], or IL-15 [18] can be used for NK cell activation and expansion in culture and give rise to effector cells with potent anti-tumor capacity [16] that have been tested in phase 1 clinical trials [19, 20] with conflicting results. Alternatively, adoptively transferred NK cells could be combined with a monoclonal antibody specific to AML blasts, such as CD33, to augment ADCC. Due to the transient persistence of donor NK cells and monoclonal antibody, long-term myelotoxicity with this therapy is theoretically less likely to occur than with CAR-T cells [21]. Together, the therapeutic usefulness and clinical applications of ex vivo modified NK cells is promising but remains to be determined.

Although the number of KIR–HLA mismatched NK cells was not associated with survival, certain cell surface markers were and could be used as biomarker to predict individual responses to NK cell therapy in the future. However, further validation of these markers in clinical trials is needed.

Collectively, this phase 2 study was designed to assess the efficacy of adoptive immunotherapy with haploidentical and KIR–HLA-mismatched NK cells. Despite tolerability and transient engraftment of donor NK cells, adoptive transfer of NK cells did not improve event-free or overall survival rates in children with intermediate-risk AML. Nevertheless, our results do not preclude the potential benefit of NK cells in children with active disease, as this was demonstrated to be effective in adult studies [7, 22–24]. Given the relatively favorable toxicity profile, repeated infusions of NK cells are possible and may reduce tumor burden, thereby inducing remission during earlier phases of treatment. Alternatively, repeated NK cell infusions during maintenance therapy in the setting of remission could provide ongoing immune surveillance and may be tested in future clinical trials.
